# Calendar

**DOI:** 10.1155/S146392468000051X

**Published:** 1980


### Clinical analyser

A second generation discrete analyser is now available from Vitatron. The PA800 is a micro-computer based analyser on which both large batches and small numbers of samples can be run. A non-primed dispensing system means rapid test changeover and this, coupled with simplicity of operation, allows one operator to run all the analyses. It also allows the operator enough time on large batches of samples to perform other jobs round the laboratory. Additional features include full programming flexibility, small sample and reagent volumes and low running costs.

*MSE Scientific Instruments, Manor Royal, Crawley RH10 2QQ, UK.*

### Computing integrator

A computing integrator for gas and liquid chromatography has been introduced by Pye Unicam. The CDP1 computing integrator is designed around an 'easy key' control giving rapid familiarisation and simple operation. It is available with or without a digital display and has a built-in printer which outputs parameters, peak retention times and areas or heights, with baseline codes, followed by the calculated result.

A choice of manual, semi-automatic and automatic methods for peak detection ensures accurate area allocation throughout the analysis. A full self-integrity check with built-in signal generation to check correct integration, is also incorporated. Three built-in calculation methods are available, area % normalisation, normalisation with a scale factor, and an internal standard method.

*Pye Unicam Ltd, York St, Cambridge CB1 2PX, UK.*

### Calendar

**Editor's Note:**

***Organisers of conferences, seminars etc. should send details for inclusion in this calandar as soon as the relevant information is available and not later than three months before the event.***

**3rd International EPR Symposium**
August 11-14, Denver, U.S.A.
*Gareth R. Eaton, Department of Chemistry, University of Denver, Denver, Colorado, U.S.A.*

**Gordon Research Conference on Analytical Chemistry**

August 11-15, New Hampton.

*Gordon Research Conferences, Colby-Sawyer College, New London, NH 03257, U.S.A.*

**2nd Chemical Conference of the North American Continent**
August 24-29, San Francisco.
*A.P. Winstead, American Chemical Society 1155 16th St. NW, Washington, DC 20036, U.S.A.*

**7th European Congress on Electron Microscopy**

August 24-30, The Hague.

*Laboratory for Electron Microscopy, University of Leyden, Rijnsburgerweg 10, Leyden, The Netherlands.*

**Measurement and control of chemical hazards in the workplace environment**
August 25-28, San Francisco.
*Gangadhar Choudhary, National Institute for Occupational Safety and Health, DPSE, Mail Stop R3 4676 Columbia Parkway, Cincinnati, Ohio 45226, U.S.A.*

**12th Australian Spectroscopy Conference**

August 25-29, Sydney, Australia.

*Professor B.S. Orr, School of Chemistry, The University of New South Wales, PO Box 1, Kensington, NSW, Australia 2033.*

**11th International Congress of Clinical Chemistry — 4th European Congress of Clinical Chemistry**
August 30-September 5, Vienna
*Congress Secretariat, Interconvention, PO Box 35, A-1095 Vienna, Austria.*

**Immunoassay workshop**
August 31-September 5, Guildford, U.K.
*Courses Secretary, Department of Biochemistry, University of Surrey, Stag Hill, Guildford GU2 5XH, U.K.*

**Annual meeting of the British Association for the Advancement of Science**

September 1-5, Salford.

*Miss J.H. Dring, BAAS, 23 Saville Row, London W1, U.K.*

**Laboratory methods and techniques**
September 2-4, Portsmouth.
*J.M. Potts, Room 306, Chemistry Dept, St Michael's Building, Portsmouth Polytechnic, White Swan Road, Portsmouth PO1 2DT, U.K.*

**12th Annual Conference of the European Group for Atomic Spectroscopy**
September 2-5, Pisa.
*A. Gozzini, Instituto di Fisica, Universita di Pisa, Piazza Torricelli 2, 56100 Pisa, Italy.*

**International solvent extraction conference**
September 6-12, Liege, Belgium.
*Conference Secretary, ISEC 80, Dept of Chemistry, University of Liege, Sart Tilman, B-4000 Liege, Belgium.*

**3rd World Filtration Congress**

September 13-17, Philadelphia.

*Filtration Society, Shippensburg, Philadelphia, U.S.A.*

**Modern techniques in centrifugation**

September 14-19, Colchester.

*Liaison Officer, University of Essex, Wivenhow Park, Colchester CO4 3SQ, U.K.*

**6th International Symposium on Advances and Applications of Chromatography in Industry**
September 16-19, Bratislava, Czechoslovakia.
*Jan Remen, Analytical Section CS VTS, pri np Slovnaft, 82300, Bratislava, Czechoslovakia.*

**Clinical laboratory automation**
September 19-20, Tokyo.
*Japanese Society of Clinical Laboratory Automation, 9-3 Nakamarucho, Itabashi-ku, Tokyo 173, Japan.*

**High performance liquid chromatography course**
September 22-26, Loughborough.
*Miss J.M. Brown, Department of Chemistry, Loughborough University of Technology, Loughborough, Leics. LE11 3TU, U.K.*

**Trace and ultratrace analysis**
September 23-25, Cardiff.
*The Secretary, Analytical Division, Chemical Society, Burlington House, London W1V 0BN, U.K.*

**7th Annual meeting of the Federation of Analytical Chemistry and Spectroscopy Societies**
September 28-October 3, Philadelphia.
*S.W. Fleming, Engineering Physics Laboratory, Du Pont Experimental Station, Wilmington, Del 19898, U.S.A.*

**Analysis 80 — The installation and management of micro and mini computers in the laboratory**
September 29-30, London.
*Scientific Symposia Ltd, 33-35 Bowling Green Lane, London EC1R 0DA, U.K.*

**ASTM E-19 Meeting on the Theory and Practice of Chromatography**
October 1-3, Pennsylvania State University.
*Clayton O. Ruud, 159 Materials Research Laboratory, Pennsylvania State University, University Park, Pa 16892, U.S.A.*

**INTERKAMA 80 – 8th International Congress and Exhibition for Instrumentation and Automation**
October 9-15, Dusseldorf, West Germany.
*Arbeitsgemeinschaft INTERKAMA, Postfach 700969, D-6000 Frankfurt/M 70, Federal Republic of Germany.*

**Pollution analysis and control**
October 14-16, Stoke-on-Trent.
*Assistant Secretary, British Ceramic Society, Shelton House, Stoke Road, Shelton, Stoke-on-Trent S14 1DR, U.K.*

**New spectroscopic methods for biomedical research**
October 20-21, Columbus, Ohio.
*Karen Waite, Battell-Columbus Laboratories, 505 King Ave, Columbus, Ohio 43201, U.S.A.*

**Multidiscipline Spectroscopy Symposium**
October 27-31, Chicago.
*Donald H. Arendt, Wells Manufacturing Co, 7800 N Austin Ave, Skokie, Illinois 60077, U.S.A.*

**Electronica 80**
November 6-12, Munich.
*Munchener Messe und Ausstellungsgesellschaft mbH, Postfach 12 10 09, D-8000 Munchen 12, Federal Republic of Germany.*

**MAC '80 – 20th International Exhibition of Chemical Analysis, Research and Test Equipment**
November 11-15, Milan, Italy.
*MAC – Via Domenichino 11, 20149, Milano, Italy.*

**1st African and Mediterranean Congress of Clinical Chemistry**
November 11-15, Milan, Italy.
*1st African and Mediterranean Congress of Clinical Chemistry, Via Keplero 10, 20124 Milan, Italy.*

**New directions in automation in the clinical laboratory**
November 17-18, Dusseldorf.
*Robert S. First Inc, 19a Avenue Marnix, 1050 Brussels, Belgium.*

**International symposium on application of microprocessors in devices for instrumentation and automatic control**
November 17-20, London.
*IMEKO/IFAC Symposium Organising Committee, The Institute of Measurement and Control, 20 Peel Street, London W8 7PD, U.K.*

**Electro-analytical techniques – industrial and clinical applications**
November 25-27, London.
*Sira Institute Ltd, South Hill, Chislehurst, Kent BR7 5EH, U.K.*

### 1981

**Biotechnology – second European Congress**
April 5-10, Eastbourne
*Secretariat, ECB2, c/o Society of Chemical Industry, 14 Belgrave Square, London SW1 8PS, U.K.*

**Euroanalysis IV**
August 23-28, Helsinki, Finland
*Association of Finnish Chemical Societies, Executive Secretary, Pohj, Hesperiankatu 3B10, SF-oo260 Helsinki 26, Finland*

### 1982

**International Congress on Automation in the Clinical Laboratory**
April 19-22, Barcelona, Spain.
*Dr. R. Galimany, Departmento de Analisis Clinicos, Seccion de Automatizacion, Cuidad Sanitaria 'Principes de Espana', Hospitalet de Llobrogat, Barcelona, Spain.*

